# Common Factors of Alzheimer’s Disease and Rheumatoid Arthritis—Pathomechanism and Treatment

**DOI:** 10.3390/molecules26196038

**Published:** 2021-10-05

**Authors:** Paulina Trzeciak, Mariola Herbet, Jarosław Dudka

**Affiliations:** Chair and Department of Toxicology, Faculty of Pharmacy, Medical University of Lublin, Jaczewskiego 8b Street, 20-090 Lublin, Poland; trzeciakp@onet.pl (P.T.);

**Keywords:** Alzheimer’s disease, rheumatoid arthritis, amyloid, immune system

## Abstract

The accumulation of amyloid plaques, or misfolded fragments of proteins, leads to the development of a condition known as amyloidosis, which is clinically recognized as a systemic disease. Amyloidosis plays a special role in the pathogenesis of neurodegenerative diseases such as Alzheimer’s disease (AD), Parkinson’s disease, and rheumatoid arthritis (RA). The occurrence of amyloidosis correlates with the aging process of the organism, and since nowadays, old age is determined by the comfort of functioning and the elimination of unpleasant disease symptoms in the elderly, exposure to this subject is justified. In Alzheimer’s disease, amyloid plaques negatively affect glutaminergic and cholinergic transmission and loss of sympathetic protein, while in RA, amyloids stimulated by the activity of the immune system affect the degradation of the osteoarticular bond. The following monograph draws attention to the over-reactivity of the immune system in AD and RA, describes the functionality of the blood–brain barrier as an intermediary medium between RA and AD, and indicates the direction of research to date, focusing on determining the relationship and the cause–effect link between these disorders. The paper presents possible directions for the treatment of amyloidosis, with particular emphasis on innovative therapies.

## 1. Amyloid Plaques, Structure, Importance, Factors Predisposing to Their Appearance

Amyloidosis is a disease associated with the extracellular accumulation of misfolded protein fragments [1]. Amyloid proteins are characterized as “chameleon proteins” due to their characteristic ability to adopt several conformations [2]. It should be noted that all amyloid proteins are unbranched and have a diameter of 70 to 120 Å [3]. The first studies on isolated amyloid fibrils concerned the knowledge of their structure. X-ray diffraction studies have shown that amyloid-like proteins have a cross-structure β [4,5]. Later studies using NMR analysis further confirmed this hypothesis [6]. Due to the ubiquitous presence of proteins in the body’s cells, amyloidosis is clinically considered a systemic disease [7]. For example, amyloid accumulation plays a special role in the pathogenesis of Alzheimer disease (AD), Parkinson’s disease, or transthyretin amyloidosis [8]. Protein abnormal folding, characteristic of amyloidosis, most often concerns the transthyretin protein (TRT) and the immunoglobulin light chain [9].

In amyloidosis, a protein acquires an abnormal structure through a variety of mechanisms. One of them is that the protein has a naturally intrinsic tendency to adopt a pathological structure that becomes visible with age. The present situation occurs in senile systemic amyloidosis [10]. A separate mechanism is that substitution results in replacing a single amino acid with another, which predisposes to the emergence of hereditary amyloidosis [11]. The last mechanism concerns the proteolytic remodeling of the precursor protein [12].

Amyloid is a structure that occurs in the form of insoluble and pathological deposits. The most common component of the deposits is the SAP (serum amyloid P) glycoprotein, belonging to the pentraxin family, which after radioactive labeling becomes a diagnostic tool for imaging the presence of pathological proteins [13]. The SAP glycoprotein is protected against proteolysis, and this property ensures its resistance to degradation [14]. Proteoglycans are also a component of amyloid that exhibit similar cell deposition kinetics as fibrillar proteins. Moreover, they are located in the vicinity of specific structures of the extracellular matrix such as perlekan, laminin, entactin, and collagen IV [15].

Amyloid accumulation is dependent on several factors. High local protein concentration, low pH in the cell, and the presence of filamentous seeds predispose to the accumulation of pathological deposits. Amyloid deposition is additionally influenced by the presence of specific receptors on the cell surface. RAGE receptors are such receptors—the receptor for advanced glycation end-products, which is the receptor for A-β [16].

The aim of the current research work is to show how amyloid contributes to tissue damage and organ dysfunction. The most common theory explores that the presence of amyloid conglomerates disrupts the proper functioning of structures in its vicinity. The pressure exerted by the amyloid becomes a direct cause of degeneration of the tissues and organs in the vicinity of the amyloid. It is worth mentioning that the accumulation of proteins with an abnormal structure leads to the disturbance of the organism’s homeostasis [17].

The initiation process of amyloid formation is related to the human biological clock (especially with old age), genetic mutations, modifications after protein synthesis, or it results from an increased concentration of amyloidogenic precursor [18]. Many scientific works emphasize the search for links between the aging process of the body and the occurrence of amyloidosis. It is known that the risk of developing amyloidosis increases with age. The aging of the organism predisposes to the appearance of amyloid in tissues and organs and is also a signal for the deposition of proteins with a pathological structure [19]. The above dependence is related to the importance of the protostatic process, which fluctuates with age. The main importance of this process lies in maintaining the proper concentration of proteins in cells, their spatial structure, and their subcellular localization [20]. It should be pointed out that the aging of the organism initiates the deposition of amyloid precursor proteins such as amyloid β (Aβ), tau, or α-synuclein, the presence of which is associated with the pathology of Alzheimer’s or Parkinson’s disease [21]. Furthermore, one must mention that for the fibrillation process, it is necessary to have a sufficient amyloidogenic potential of the protein and the achievement of a critical local concentration by the protein precursor. The initiation process can be enhanced by the above-mentioned factors and the interactions of the protein with extracellular matrices [22].

The most commonly deposited pathological proteins are transthyretin (TRT), islet amyloid polypeptide (IAPP), atrial natriuretic factor (ANF), apolipoprotein AI (ApoAI), and the recently discovered fibulin-like substance containing epidermal growth factor: extracellular matrix protein 1 (EFEMP1) in the extracellular matrix1 (EFEMP1) people, APrP (Prion protein, wild type), ACal ((Pro)calcitonin), ASem1, Semenogelin 1, and huntingtin [23,24]. The authors resigned from a detailed discussion of all pathological proteins. However, it should be noted that AD and RA discussed in the work are diseases with amyloid pathology, which required an introduction to this topic. Moreover, a lot of previous scientific works on amyloidosis have mainly focused on the pathology of amyloid in Alzheimer’s disease [25]. However, in the field of recent research, it has been noted that amyloidosis is a disorder involving many tissues and organs. In these studies, the existence of cause–effect relationships between amyloid accumulation and the occurrence of metabolic diseases, cardiovascular diseases, and skeletal system disorders has been reported [26] (Table 1). The main determinant of research on amyloid is the need to determine the relationship between the aging process of the body and the predisposition to the accumulation of pathological proteins. Exploring similar topics is justified, since life expectancy is longer, and nowadays, old age is determined by the comfort of functioning or the elimination of unpleasant symptoms of diseases accompanying the elderly [27].

Amyloid accumulation takes place throughout the body: in the central and peripheral nervous system and in many organs: the heart, kidneys, and the bone and joint system [29]. The literature describes many types of systemic amyloidosis, four of which are seen most frequently: AL (immunoglobulin light chain amyloidosis), AA (also known as secondary amyloidosis), ATTR (transthyretin amyloidosis), and Aβ2M (Beta-2 Microglobulin amyloidosis) [30]. These amyloidoses are described in Table 2.

## 2. Amyloid Plaques in AD and RA

AD is a disorder of the nervous system, and according to the estimates of the World Report on Alzheimer’s disease, in 2050, its worldwide incidence will exceed 152 million people [35]. Alzheimer’s disease is a neurodegenerative disease [36]. According to the ICD-10 classification, it is defined as a progressive disorder characterized by the degradation of nerve cells [37]. Loss of neurons in memory regions of the brain causes dementia-like changes. Neurodegeneration correlates with the deterioration of cognitive functions, which, with the progression of the disease, significantly limits the efficient functioning of patients. Moreover, changes in the upper levels of the central nervous system (CNS) cause changes in the patient’s behavior or the emergence of psychiatric disorders [38].

In terms of neurophysiology, Alzheimer’s disease is associated with the appearance of two pathological structures in the brain tissue structures: extracellular amyloid plaques and intracellular neurofibrillary tangles (NFTs). The appearance of the above structures contributes to the occurrence of nerve cell atrophy [39,40].

The process of Aβ peptide formation, which is a disease marker, is related to the enzymatic cleavage of the amyloid precursor protein (APP) [41]. APP alternates in two ways, with two different cutting paths. In the non-amyloidogenic pathway, APP is cleaved by α- and γ-secretase to form Aβ17-40/42 peptide or Aβ1-16 peptide. On the other hand, in case of the amyloidogenic pathway, APP is cleaved sequentially by β- and γ-secretases, leading to the formation of full-length β peptide A (mainly Aβ1-14/42) [42]. Although Aβ1-40 is present in much greater amounts in the brain, Aβ1-42 is a less soluble form and is more prone to accumulate. The accumulation process leads to the formation of conglomerates, which are referred to in the literature as oligomers. The above structures are rearranged into protofibrils and filaments, having their organization in amyloid plaques [43]. One hypothesis is that soluble fibril precursors adopt a specific quaternary conformation that exhibits significant cytotoxicity that is largely unknown at present. Cellular toxicity is dictated by the stimulation of oxidative stress mechanisms and additionally assumes the activation of cellular apoptotic pathways. Moreover, the aforementioned hypothesis indicates that mature, fibrillar amyloid deposits are inactive reservoir structures in balance with less toxic syndromes [44].

The presence of pathological plaques affects the neurotransmitter systems, especially the glutamatergic system. In this system, the main neurotransmitter is glutamate, which plays a key role in the processes of creating memory engrams. The activity of glutamate comes down to its mediation in learning and memory processes. The activity of glutamate is related to the second type of messenger, calcium ions (Ca^2+^), which help to create the chemical environment necessary for information gathering [45]. Under pathological conditions, excess glutamate causes an excessive intracellular influx of calcium ions, which in turn leads to calcium overload. In the environment of excessive calcium presence, nerve cells die [46]. In Alzheimer’s disease, Aβ plaques cause extracellular accumulation of glutamate and intracellular deposition of calcium ions. Non-fibrillar oligomers, which are likely present in higher concentrations in the immediate vicinity of amyloid plaques, can also disrupt calcium homeostasis [47,48]. Therefore, it is worth noting that Aβ plaques increase the susceptibility of neurons to excitotoxicity and loss of synaptic protein [49].

In Alzheimer’s disease, the dysfunction of cholinergic transmission in the forebrain is also observed. In patients with Alzheimer’s disease, the depleted presynaptic presence of cholinergic markers has been detected in the areas of the cerebral cortex, and it has been shown that the Meynert basal nucleus (NBM) located in the basal forebrain undergoes neurodegeneration as the disease progresses [50,51]. The loss of neurons in the forebrain and limbic system leads to dysfunctional changes in nicotinic receptors with a decrease in their density in the cerebral cortex and influences the activity of muscarinic receptors in the cerebral cortex [52,53].

The cholinergic neurons of the forebrain are the cells with the greatest neurodegenerative potential and also the structures most susceptible to the formation of neurofibrillary tangles [54]. The impoverishment of cholinergic transmission is caused by the presence of amyloid, and this relationship correlates with the negative effect of senile plaques on choline acetyltransferase, which participates in the synthesis of acetylcholine [55]. Studies in animal models have shown that cholinergic loss results in increased accumulation of Aβ and tau protein [56]. On the basis of other studies, it is determined that disturbances of cholinergic transmission in the brains of rats induce pro-inflammatory mechanisms and influence the disclosure of cognitive disorders [57]. Acetylcholine, being a neurotransmitter of the cholinergic system, additionally affects the functionality of the blood–brain barrier. It has been argued that the loss of cholinergic transmission potentially contributes to abnormalities in the diffusion and transport of metabolites between the interstitial fluid and the cerebrospinal fluid. Impairment of substance exchange across the blood–brain barrier impairs the clearance of Aβ from the brain [58]. It has also been shown that defective cholinergic transmission affects the continuity of the blood–brain barrier and thus disrupts the perivascular clearance of Aβ [59].

It should be noted that the accumulation of amyloid beta begins in other parts of the brain. On the basis of the research of Palmqvist et al. [60], it is known that the accumulation of Aβ fibrils begins in certain regions of the brain, before they can be found throughout the neocortex, and before neurodegeneration is present. The researchers described that the early stages of amyloid deposition take place in the precuneus, posterior cingulate cortex, and orbitofrontal cortex. When examining subjects with even earlier signs of Aβ accumulation (CSF (cerebrospinal fluid)−/PET (positron emission tomography)− subjects who converted to CSF+/PET− within 2 years), a significantly increased Aβ fibril accumulation rate was again seen in the medial orbitofrontal and posterior cingulate cortex compared with stable CSF−/PET− subjects [60]. Braak [61] described the tau pathology progression from locus coeruleus through the transentorhinal region to cortical areas. These data suggest that tauopathy associated with sporadic Alzheimer disease may begin earlier than previously thought and possibly in the lower brainstem rather than in the transentorhinal region [61].

In recent years, scientific works examining the influence of peripheral processes on the pathomechanism of nervous system diseases have gained great value. In AD, it refers to the influence of the immune system on the onset of the disorder, which is the inflammatory basis of the disease. Thus, according to the literature, immunological mechanisms are responsible for the occurrence of dementia-like disorders [62]. The inflammatory reaction within the central nervous system is mediated by microglial cells. The activated microglia produce pro-inflammatory cytokines such as TNF-α (tumor necrosis factor-α), IL-1 (interleukin-1), IL-6 (interleukin-6), and chemokines. The above mediators of the inflammatory reaction initiate the process of chronic inflammation within the nervous system, leading to the death of neurons and the intensification of neurodegenerative changes [63,64,65]. The relationship of Aβ with the inflammatory theory is justified because senile plaques intensify the production of free radicals and initiate the cascade of inflammatory processes [66]. This is due to the presence of microglia near senile plaques and neurons containing neurofibrillary tangles, which, when mobilized, constitute an immunological line of defense [67,68]. Mobilized microglia can show a twofold phenotype depending on the environment. The protective function of microglia becomes apparent when Aβ fibers or neuronal debris are removed or when it is involved in synaptic remodeling and the release of growth factors. In contrast, the neurotoxic phenotype stimulates the activity of cytokines such as TNF-α and IL-1β (interleukin 1β). These immune system mediators cause tissue damage and disease progression [69].

The accumulation of Aβ starts in the preclinical phase of AD, leading to the progression of degenerative changes within ten years. In the prodromal phase of the disease, amyloid activity shows a potential for a plateau. At this time, mild cognitive impairment (MCI) becomes apparent [70]. In the prodromal phase of the disease, hyperphosphorylation of the tau protein occurs alongside its aggregation into neurofibrillary tangles. This response contributes to the direct neuronal dysfunction and contributes to the progression of dementia and progressive dementia [71].

RA is defined as a degenerative disease of the skeletal system. The disorder is characterized by the presence of a systemic inflammatory reaction that affects articular cartilage and bones [72]. Normally, the disease process involves immune cells that cascade to engage subsequent cells and mediators. Involvement of the immune system is associated with the activation of appropriate cells, resulting in the release of matrix metalloproteinases (MMPs) and inflammatory cytokines [73]. Ultimately, the ongoing processes result in the occurrence of painful joint swelling and impairment of their functions [74].

The factors that predispose to RA are not clearly explained. It has been suggested that the disease is related to genetic and exogenous factors. The first cells involved in the pathogenesis of RA are CD4+ lymphocytes. These cells recognize antigens in the synovial tissue and stimulate monocytes, macrophages, and synovial fibroblasts. The above-mentioned cells secrete metalloproteinases that are involved in the erosion of cartilage and bone. In addition to their degradative activity, these immune cells are involved in the production of interleukin (IL)-1, IL-6, and TNF-α, which are responsible for the key inflammatory response in RA. The entire cascade process eventually leads to synovitis, which becomes thickened and enlarged [75].

Appropriate biomarkers appear in the serum in RA patients, such as rheumatoid factor (RF), anti-cyclic citrullinated peptide (anti-CCP), C-reactive protein (CRP), amyloid A protein, and calgranulin [76]. The presence of amyloid structures is of particular interest, since the amyloidosis of the light chains of transthyretin and immunoglobulins contributes to amyloid deposition in soft tissues. The accumulation process is, in effect, a direct cause of orthopedic diseases [77]. One of the studies indicated the presence of amyloid deposits detected during orthopedic procedures, especially in people over 70 years of age [78]. Moreover, a study by Donelly et al. [79] indicates that 10% of men over 50 and women over 60 after biopsy have tendon sheath positive markers of amyloid tissue [79].

Protein amyloid A (SAA, serum amyloid A), which is produced by liver cells, deserves additional attention. Hepatocytes that are stimulated by the presence of pro-inflammatory cytokines (including TNF-α, IL-1, and IL-6) produce SAA in increased amounts. One study determined that the overproduction of SAA can occur in a number of diseases related to inflammatory pathology. Such diseases include long-term inflammation, including chronic infections, such as tuberculosis, osteomyelitis and rheumatoid arthritis, inflammatory bowel disease, hereditary diseases, and hematological and solid neoplasms [80]. It is worth noting that the overproduction of pro-inflammatory cytokines promotes increased angiogenesis, the processes of connective-tissue binder degradation in RA, and increased amyloid activity, which characterize cytokines as pleiotropic mediators [81].

## 3. The Activity of the Immune System in AD and RA

Chronic systemic peripheral inflammation influences the neurodegenerative processes that are characteristic of AD. The activity of inflammatory cytokines such as TNF-α, IL-6, IL-1β, transforming growth factor beta (TGF-β), IL-12, and IL-18 is clearly noticeable in AD patients compared to healthy controls [82]. The above-mentioned cytokines and their influence are being studied both in the pathogenesis of AD and in RA, because the over-reactivity of the immune system is a common feature of these disorders.

When determining the positive correlation between AD and RA, it should be noted that the incidence of AD is much higher in RA patients than in healthy people [83]. In a separate study, cognitive decline was observed later in life in people struggling with arthritic diseases, especially RA [84]. Work on the effects of systemic inflammation has been studied through the activity of anti-inflammatory drugs. Methotrexate and non-steroidal anti-inflammatory drugs (NSAIDs), which are common pharmaceuticals used in RA, reduce the risk of AD-related dementia, especially when these drugs are administered early in the disease [85,86].

It is known that overactivity of the immune system is the bridge between these diseases; however, further research is needed to determine the exact correlation between RA and AD. A common feature between RA and AD is the dysregulation of cell cycle suppression genes, which in turn contributes to the incidence of systemic inflammation. Studies show that inflammatory changes affect the appearance of pathological changes in both disorders, and cell cycle modifications are significantly age-regulated [87].

In the context of Alzheimer’s disease, it is claimed that systemic inflammation influences the appearance of neurodegenerative changes [82]. Activation of the immune system contributes to the development of dysfunction in the central nervous system. Imaging tests show a reduced brain volume and pathological changes in white matter [88]. Systemic inflammation also affects the limited distribution of blood in the blood vessels, which contributes to brain dysfunction due to restricted oxygen supply. Therefore, it can be assumed that changes in circulating blood flow increase the risk of developing dementia [89]. In patients with depression and AD, raised levels of pro-inflammatory cytokines, acute phase proteins (APPs), interferon gamma (IFN-γ), interleukin 1 (IL-1), IL-6, and TNF-α were observed [90].

Based on the literature, it is suggested that NSAIDs (non-steroidal anti-inflammatory drugs) inhibit the formation of fibrous Aβ by stimulating α-secretase. As a result of such activity, APP is transformed by a non-amloidogenic path, which leads to a reduction in the formation of amyloidogenic forms [91]. The legitimacy of the use of NSAIDs in AD has been determined in several studies. A meta-analysis was performed, which included 16 cohort studies, conducted between 1995 and 2016 and involving 236,022 participants [92]. It showed that the current or previous use of NSAIDs was significantly associated with a reduced risk of AD compared to those who did not use NSAIDs. The study also shows correlations related to where the patients live (eight studies looked at populations from North America, six studies were from European countries, and two studies focused on Asian populations). Noteworthy, in this study, the researchers have studied the influence of the environmental factor. The authors showed that NSAID exposure was associated with a reduced risk of AD in population-based studies. Such correlations were not noted in the study on the importance of the environmental factors. The study pointed to a significant impact of NSAID exposure in Europe. The borderline effect of NSAID exposure was observed in studies conducted in North America, and NSAID exposure was not associated with the reduced risk of AD in studies conducted in Asia. Current evidence suggests an effect of NSAIDs on a reduced risk of AD especially in large population-based cohort studies. The authors suggest that there is a need for a much larger study to justify the dose of the drug and the duration of NSAID exposure [92]. Among the similar meta-analyses cited by the authors of the study is a meta-analysis performed by Etminan et al. The authors of this study report a positive correlation between the use of NSAIDs and the onset of AD. Despite the fact that it includes nine observational studies, it is necessary to point out some limitations related, among other things, to the use of a specific NSAID and the reduction of the risk of AD [93].

One study evaluated the safety and efficacy of low doses of naproxen in preventing the progression of pre-asymptomatic AD among at-risk individuals with cognitive impairment. This two-year study involved 195 elderly people (mean age 63 years) who had a positive family history of AD. Patients underwent an imaging examination to exclude cognitive impairment. The researchers have divided the study participants into two groups. One of them received a placebo, while the second group was administrated with naproxen sodium at a dose of 220 mg twice daily. Multimodal, neurosensory, cognitive imaging, and evaluation of cerebrospinal fluid biomarkers was performed at the start of the study, 3, 12, and 24 months. People who took naproxen sodium experienced side effects. Naproxen did not reduce the progression of AD. Moreover, secondary analyses did not show any significant effect of treatment on individual biomarker indicators of cerebrospinal fluid nor cognitive or neurosensory progressive pre-symptomatic AD [94]. The effectiveness of NSAIDs in AD was studied on the example of naproxen and celecoxib. This was a large, multi-year randomized, placebo-controlled clinical trial. The researchers concluded that not every drug can delay the onset of AD in adults with a family history of dementia [95].

The implementation of appropriate treatment for the patient often depends on their economic status. Particularly noteworthy is the study conducted by Moilna et al [96]. The aim of the study was to determine the relationship between socioeconomic status (SES) and delays in treatment with disease-modifying antirheumatic drugs (DMARDs) in RA patients. A total of 1209 RA patients were recruited for the study, of whom 1159 received DMARD treatment. Researchers have assessed SES based on education, occupation, and income. After a preliminary analysis, they divided the patients into terciles. The researchers assessed the severity of the disease, which was determined by the rate of disease activity in 28 joints. They determined joint damage based on manual radiographs according to the Sharp scale and physical disability on the basis of a modified Health Assessment Questionnaire (M-HAQ). The researchers conclude that patients with lower SES experience longer delays in starting DMARD treatment. Additionally, patients who experience a greater delay in initiating DMARD treatment do experience a clinically significant increase in joint damage [96].

The relationship between AD and RA was investigated by Chou et al. [97]. The authors of the study analyzed medical and pharmaceutical claims. The analysis took into account data from adults who were commercially insured between 2000 and 2007. After analyzing the data, 325 people showed symptoms of AD and were diagnosed with RA. The study took into account the presence of concomitant diseases in patients such as coronary artery disease, diabetes, hyperlipidemia, hypertension, and peripheral vascular disease. It was determined that the overall incidence of AD among people with RA was 0.79%, while the incidence of AD among those without RA was 0.11%. In addition, it was shown that chronic conditions such as coronary artery disease, diabetes, and vascular disease significantly increased the relative risk of AD among patients with RA. The authors of the study additionally analyzed patients suffering from RA. For this purpose, the prevalence of AD among patients with RA after exposure to various RA therapies was determined. The drugs used in RA were methotrexate, prednisone, sulfasalazine, three anti-TNF drugs (adalimumab, etanercept, and infliximab) and an anti-CD20 drug (rituximab). The researchers concluded that anti-TNF therapy in RA lowered the risk of AD among these patients. Although the researchers have shown that inflammation is probably important in the pathology of AD, more research should be carried out in this direction [97]. Ungprasert et al. [98] drew similar conclusions and indicated an increased risk of dementia in patients suffering from RA [98]. The authors of the study suggest that the correlation between AD and RA should be linked to blood vessel disease. In the above monograph, the authors indicate that atherosclerosis caused by RA is a possible explanation for such a correlation. Atherosclerotic cardiovascular disease is a well-known risk factor for dementia [99]. In addition, the predisposition to the occurrence of atherosclerosis in patients with RA results from the negative impact of intract cytokines on vascular endothelial cells [100]. It is worth noting that the occurrence of cardiovascular diseases is more common among patients with autoimmune inflammatory diseases [101].

Mendel’s randomized controlled trials assessing the potential relationship between AD and RA are of particular interest. The authors of the study used publicly available collections of studies consisting of three-stage trans-ethnic, European-specific, and Asian-specific genome-wide association study (GWAS) meta-analyses, which evaluated 10 million single nucleotide polymorphisms (SNPs) in 29,880 RA cases and 73,758 controls assigned as exposures. The researchers used Mendelian randomization (MR), which uses genetic variants as instrumental variables. Its use assesses the relationship between the risk factor or exposure and whether the outcome is consistent with the causal effect. The analysis is dedicated to studies in which it is difficult to measure exposure and outcome in the same group of people. The aim of the study was to determine whether RA is causally related to AD using MR analysis of two trials. In this study, Bae and Lee [102] indicated a significant causal relationship between RA and AD. The researchers suggest that the study has some limitations. First of all, genetic variants have little effect on RA, and there is a need to experiment on a large population of people. In addition, the work was carried out on the European population. This is an inaccurate fact, as the relationship between AD and RA may be related to ethnicity. Therefore, the authors suggest that there is a need to conduct a study on a diverse study group. [102].

Other researchers have noted that drug therapy with anti-rheumatic drugs reduces the risk of dementia [103]. Researchers observed a reduced risk of dementia in cDMARD users compared with those not taking such medications. In addition, after the use of drugs, there was a reduction in the risk of dementia in those using cDMARD. The strongest effect was achieved with the use of MTX (metotrexat). The risk of dementia was 0.5% compared to 1.6% at 5 years and 1.5% at 3.0% at 15 years. The results obtained by the researchers are consistent with the estimated incidence of dementia in the UK in 2013, which was 1.3%. Despite the results, the authors of the study point to some limitations. The study involved people who suffered from comorbidities, including cardiovascular disease, myocardial infarction, congeal heart failure, peripheral vascular disease, interstitial lung disease, anemia, and osteoporosis. Before starting RA therapy, patients took antihypertensive drugs, analgesics, statins, or proton pump inhibitors. [103].

One of the newest studies conducted in 2020 confirms the above-mentioned findings and additionally indicates the potential protective effect of anti-TNF drugs in patients with RA [104]. This study was conducted in 56 million adults in the United States and showed a positive correlation between AD and RA and a lower risk of AD in patients taking etanaercept, adalimumab, or infliximab. The authors of the study indicate that the above-mentioned biological drugs have poor distribution to the brain, and by acting systemically, they prevent TNF-α from entering the brain and thus prevent the risk of dementia. Thanks to this research, it is estimated that treatment of RA with TNF inhibitors can help prevent about 4.0% of AD cases (0.21 million) [104].

The correlations between AD and RA were investigated by McGeer and others [105]. Seventeen epidemiological studies from nine countries were used for the review. All these studies concluded that arthritis is a predisposing factor to the appearance of AD. The analysis also included studies that concluded about the positive importance of anti-inflammatory drug therapy in AD. Using statistical meta-analysis methods, the researchers estimated the risk of developing AD in RA patients and anti-inflammatories compared to the general population. Seven case-control studies with arthritis as a risk factor yielded a total odds ratio of 0.556 (*p* < 0001), while four case-control studies with steroids yielded an odds ratio of 0.656 (*p* = 0.049). Three case-control studies with NSAIDs gave an odds ratio of 0.496 (*p* = 0.0002). When NSAIDs and steroids were combined into one category of anti-inflammatory drugs, the odds ratio was 0.556 (*p* < 0.0001). Therefore, the study confirmed the validity of the hypothesis of the protective effect of anti-inflammatory drugs in AD. Still, the researchers suggest that there is a need for clinical trials to thoroughly verify this hypothesis [105].

In turn, Kao et al. [106], in a case-control study on 2271 patients with AD and 6813 patients in the control group without AD, indicated an inverse relationship between previous AD and RA. The mean age of the study group was 76.5 ± 7.9 years, while the mean age of the control group was 76.5 ± 8.3 years. After data analysis, significant differences were identified in geographic region (*p* < 0.001), level of urbanization (*p* = 0.002), hypertension (*p* < 0.001), diabetes (*p* = 0.027), hyperlipidemia (*p* < 0.001), stroke (*p* < 0.001), and coronary heart disease (*p* < 0.001) between cases and the control group after matching the sex, age group, and year of the index date. There was no significant difference in monthly income between the cases and the control group. The study showed that the earlier onset of RA was negatively associated with AD even in people suffering from comorbidities. Despite this correlation, the authors note that the study does not take into account several factors. First, the database used by the researchers does not contain information on the presence of inflammatory markers. What is more, the database lacked family interviews and indications of possible genetic predisposition. The researchers note that such factors can affect cognitive function and falsify the result of the study. The database also has no information on biochemical tests or medical imaging. In addition, the majority of the Chinese population was recruited for the study, which does not allow generalizing the results and relating them to the general public [106].

Similar conclusions were reached by Policicchio et al. [107], who determined that RA was associated with a lower incidence of AD. Researchers have shown that there is no correlation between the use of NSAIDs and AD. The indicated meta-analysis included eight case-control studies and two population studies. The causal relationship was determined on the basis of the MR discussed earlier. Previous analysis of the literature showed that RA was associated with a lower incidence of AD. The authors of the study question this hypothesis because MR analysis showed no correlation between AD and RA. Although there is epidemiological evidence to support the validity of the claim, the authors recognize that there are no causal relationships between these disease entities. Researchers suggest that a large impact on the validity of the thesis is influenced by factors interfering with research such as selection error or differential diagnosis of RA [107].

Therefore, the literature indicates that inflammation is a common feature of both RA and dementia, and this is confirmed by common inflammatory biomarkers found in both diseases (e.g., interleukin-6, interleukin-12, C-reactive protein, penxine 3, endotheline-1, resistin, and receptors for the end products of advanced glycation) [108]. In addition, by examining the broad relationships between the occurrence of AD, significant relationships between the occurrence of AD and the over-reactivity of the immune system were indicated. These studies have indicated the genetic overlap between AD and immune-mediated diseases [109]. Despite the presence of common markers of inflammation, this mention should be treated with great caution. Perhaps the presence of markers of inflammation does not indicate a cause-and-effect correlation between these disorders. Perhaps AD and RA are governed by other mechanisms of the formation of an inflammatory reaction, and the only feature that unites them is the presence of indicated markers. This topic requires further work to explain the possible mechanisms of the formation of an immune cascade both in AD in RA and in both disorders.

## 4. The Role of the Blood–Brain Barrier in AD and RA

The effects of systemic inflammation (infection, pathological conditions, sepsis) affect tissues and organs; however, it should be noted that the blood–brain barrier (BBB) becomes the mediating factor between RA and AD. The hyperactivity of the immune system, expressed by an increased concentration of inflammatory mediators, negatively affects the structure and permeability of the barrier [110]. Based on scientific studies, barrier permeability has been shown to be altered in RA patients. Barrier dysfunction is also associated with neurodegenerative diseases, including AD [111]. Among the mediators of the immune system, inflammatory cytokines deserve special attention, as they show the ability to easily exceed the BBB [112]. The barrier is crossed with the use of various structures, that is: by passing through the periventricular organs [113], by stimulating the vagus nerve [114], and by direct binding to the endothelium, which results in the opening of tight junctions, penetration of the cytokine, and its activity within the brain tissue [82].

The relationship between RA and AD has been studied in rats with collagen-induced arthritis (CIA) [115]. This model is characterized by a clinical picture of RA. In addition to immune system activity in the disease, the integrity of the BBB was also studied by measuring the expression of Aβ transport proteins in the blood vessels of the brain. The study showed an increase in astrogliosis, the level of peripheral and brain cytokines, and activation of microglia in the brain in CIA rats compared to the control group. It should be mentioned here that astrogliosis is observed at the time of neuronal degradation in response to injury, infection, or the presence of a neurodegenerative disease. During the study, barrier permeability was measured using sodium fluorescein and found that it was significantly increased in CIA rats. The vascular changes observed were associated with increased expression of matrix metalloproteinase and decreased expression of tight junction proteins. Occludin is one such protein. Increased expression of the RAGE receptor in the hippocampus, which is involved in the influx of Aβ from the blood to the brain, was also observed during the study. To visualize the transport of Aβ via the BBB in CIA rats, animals were administered Aβ42 intravenously. It was shown that the concentrations of Aβ42 in the cortex and in the whole brain were compared between the CIA rats and the control group. However, it was shown that in the hippocampus, the Aβ level of the CIA rats was approximately 1.8 times higher than that of the control. This relationship indicates an increased influx of Aβ from the blood to the hippocampus of CIA rats [115].

It was also suggested that RA patients are a group predisposed to disturbances of homeostasis within the blood vessels, heart, and cerebral vessels. The studies were conducted in CIA rodents and investigated endothelial dysfunction induced by chronic inflammation in RA. The expression of strict proteins was determined by immunoblotting and occludin immunofluorescence. Reduced expression of the protein involved in the formation of tight junctions (occludins) has been reported. The study concludes that barrier integrity is impaired in the pathophysiology of RA [116].

## 5. Therapeutic Strategies Targeting to Aggregates or Oligomers, Which Are the Most Dangerous Amyloid Forms

The amount of discovered amyloidogenic proteins continues to increase, which makes it difficult to diagnose patients suffering from amyloidosis. In the first stages of treatment, it becomes necessary to identify the amyloidogenic protein in order to make a proper diagnosis and implement appropriate treatment. Correct diagnosis is based on the use of histochemical tests [117], biochemical tests [118], genetic analyses [119,120], and functional imaging studies. At the present stage, the most effective approach to the treatment of systemic amyloidosis is to stop or reduce the synthesis of the amyloid precursor [121]. It is indicated that disruption of the expression of the corresponding gene using antisense oligonucleotides and small interference RNA can significantly reduce the amount of amyloidogenic precursor. Such a solution had a positive effect in the form of reduction of the synthesis of amyloidogenic light chains [122]. Despite the promising effect, the application of the present method in clinical trials faces some difficulties, including modulation of the intracellular concentration of interfering RNA [123].

First innovation in the treatment of amyloidosis was achieved by inhibiting proteases, which are responsible for the generation of amyloidogenic fragments. It is estimated that this strategy would also find application in the treatment of Alzheimer’s disease. This is because the therapeutic target in AD is to inhibit β- and γ-secretases that produce the amyloidogenic peptide [120]. In addition, the new clinical approach points to the positive importance of lipid-lowering drugs from the statin group, which can prevent the progression of AD through a mechanism related to the modulation of the ability of secretases to cleave the amyloid precursor [124]. In addition, anti-inflammatory drugs used in the treatment of AD may have a direct influence on secretase activity [125]. The use of secretase inhibitors in therapy has some limitations. For example, one of the γ-secretase inhibitors, R-flurbiprofen (Flurizan ^TM^), had a limited effect on patients with mild AD and had no effect on patients with moderate AD [126]. In addition, a significant limitation of such treatment is the fact that γ-secretases take part in many physiological functions in humans. Therefore, it is an enzyme not specific to APP, because it participates in the regulation of cell growth and in the transformation of proteins. This insufficiency contributes to the toxic effects in humans [127]. This adverse effect creates harmful defects in memory processing, myelination, and motor coordination [128]. The limitation of using secretase inhibitors is also the BBB, because the endothelial cells of this barrier restrict the diffusion of large or hydrophilic molecules. The secretase inhibitor needs to cross the BBB and neuronal membranes, because the process of modification of APP runs in the endosomes of neurons within the brain. It is also known that the largest molecular size that can cross the BBB is approximately 550 DA, so in the development of new drugs, the size of the molecule is important [126]. It is also considered that for AD therapies, it is necessary to treat individuals in the “preclinical” (presymptomatic) stage [129].

Progress in science was brought by the recently described therapy with the use of mesenchymal stem cells (MSC) [130]. These cells are isolated from bone marrow, adipose tissue, and the umbilical cord [131]. They are pluripotent in nature and have the ability to transform into any type of cell: osteoblasts, chondrocytes, and adipocytes. MSCs exhibit a wide range of activities and influence the function and activity of the immune system by modulating the proliferation of key mediators of innate and acquired immunity. The injection of MSC into the brain in AD and intra-articular in RA is beneficial because inflammation is reduced, cell proliferation is stimulated, and behavioral indicators are improved [132].

Currently, great hopes are placed on aducanumab, which in amyloid PET imaging limited the pathology of amyloid and was effective at reducing dementia in patients in phase III clinical trials [133]. If subsequent assumptions are met, aducanumab may become the first drug modifying the course of the disease, thus confirming the validity of the anti-amyloid strategy [133]. Aducanumab therapy is associated with adverse reactions known as Amyloid Related Imaging Abnormalities (ARIA). During therapy, in a mice model, bleeding and swelling of the brain were observed, which consequently causes headaches, confusion, or convulsions. For comparison, Xiong et al. [134] in 2021 did not notice these adverse effects after therapy with a new antibody targeting APOE. This antibody is determined as HAE-4. In studies in mice treated with HAE-4, amyloid plaques were removed from brain tissue and blood vessels without increasing the risk of bleeding into the brain. What is more, the researchers pointed out that the brain’s blood vessels showed a greater ability to expand and narrow on demand, and inflammation within the brain was significantly reduced [134]. A monoclonal antibody targeting amyloid is also a candidate for treatment—it is BAN240, developed by Eisai and Biogen [135]. However, it is highly probable that when used alone, these agents will not be able to limit or cure such a complex pathology of amyloidosis. There is great hope in the use of combination therapy [135].

## 6. Conclusions

Systemic inflammation affects the appearance of neurodegenerative changes. The similarity of AD and RA pathomechanisms is visible in the activity of the immune system, which, under the influence of appropriate factors, produces inflammatory biomarkers (e.g., interleukin-6, interleukin-12, C-reactive protein, penxine 3, endotheline-1, resistin, and receptors for the end products of advanced glycation). Cytokines mediate many processes in the body and affect the tightness of the blood–brain barrier, including its integrity, and strictly reduce the expression of occluding-forming junctions. In addition, AD and RA are disorders associated with the pathology of amyloid. In AD, Aβ plaques increase the susceptibility of neurons to excitotoxicity, loss of synaptic protein, and cholinergic transmission, while in RA, cytokine-stimulated amyloid contributes to the degradation of the bone–joint bond. The incidence of AD is much higher in RA patients than in healthy people. The existence of a complex network of connections is explained by demonstrating the correlation between the nervous, skeletal, and immune systems as well as the aging mechanisms of the organism. Despite the presence of common factors, this mention should be treated with great caution. The presence of inflammatory markers may not clearly indicate a cause-and-effect relationship between these disorders. It is possible that AD and RA have different inflammatory mechanisms and the only trait that they have in common is the presence of the markers indicated. This topic requires further work to elucidate possible mechanisms of the immune cascade in both AD in RA and in both diseases simultaneously. Currently, the treatment of systemic amyloidoses involves stopping or reducing the amyloid precursor synthesis, inter alia, by disrupting the expression of the relevant gene using antisense oligonucleotides and small interfering RNA. Innovation in the treatment of amyloidosis has also been achieved by inhibiting proteases, which may also be effective in the treatment of AD. Innovative therapies using mesenchymal stem cells and monoclonal antibodies targeting amyloid are of particular importance. As these treatments are unsatisfactory and have numerous side effects, the search for therapeutic strategies with greater efficacy and a higher safety profile is still ongoing.

## Figures and Tables

**Table 1:** Age-dependent amyloid distribution in selected organs [28].

Systemic Organs	Amyloid
Brain	Aβ α-Synuclein Tau
Lung	Transthyretin
Heart	Transthyretin Atrial natriuretic factor
Aorta	Apolipoprotein AI
Vein	EGF-containing fibulin-like extracellular matrix protein 1
Tendons and Ligaments	Transthyretin Apolipoprotein AI
Knee joint	Transthyretin Apolipoprotein AI

**Table 2:** Basic amyloidosis.

Kind of Amyloidosis	Cause	Precursor	Clinical Manifestation	Literature
AL	clonal plasma cell dyscrasia multiple myeloma non-Hodgkin lymphoma Waldenström disease	lambda or kappa immunoglobulin free light chain	-cardiomyopathy -nephrotic syndrome -renal failure -hepatomegaly -splenomegaly -diarrhea -arthropathy -bleeding -adrenal dysfunction	[31]
AA	longstanding inflammation (rheumatoid arthritis, inflammatory bowel disease) chronic infections (e.g., tuberculosis, osteomyelitis, leprosy) hereditary autoinflammatory diseases (e.g., familial Mediterranean fever, which is called FMF)	HDL3-associated apolipoprotein SAA (serum amyloid A protein) cute phase reactant	-proteinuria (progressing to nephrotic syndrome) -loss of renal function (progressing to renal failure) -bowel involvement -splenomegaly -hepatomegaly -cardiomyopathy	[32]
ATTR	autosomal dominantly inherited point mutations of the precursor protein transthyretin (TTR)	TTR	-peripheral -autonomic neuropathy -cardiomyopathy -renal failure -eye involvement (vitreous opacities)	[33]
Aβ2M	end-stage renal disease in which highly increased serum levels of β_2_-microglobulin	β_2-_microglobulin	-shoulder pains as a first manifestation - autonomic neuropathy -gastrointestinal symptoms	[34]

## Data Availability

Not applicable.
